# Mechanistic insights of hypoglycemic components from *Polygonatum* polysaccharide

**DOI:** 10.3389/fendo.2026.1744730

**Published:** 2026-03-17

**Authors:** Zeming Ren, Sisi Chen, Zhongxiu Guo, Xiyu Mei, Yeling Tong, Yane Liu, Ziyun Gao, Xuan Chen, Guanhai Dai

**Affiliations:** 1 Institute of Basic Medicine, Zhejiang Academy of Traditional Chinese Medicine, Hangzhou, Zhejiang, China; 2 College of Pharmacy, Hangzhou Normal University, Hangzhou Zhejiang, China; 3 School of Basic Medical Sciences and Forensic Medicine, Hangzhou Medical College, Hangzhou, Zhejiang, China

**Keywords:** gluconeogenesis, hypoglycemic, leptin, *Polygonatum cyrtonema* polysaccharide III, short-chain fatty acids, type 2 diabetes mellitus

## Abstract

**Background:**

This study aimed to fractionate *Polygonatum cyrtonema* polysaccharide (PP) into distinct components, evaluate their hypoglycemic efficacy using both *in vitro* and *in vivo* models, and elucidate the mechanisms of the most active fraction.

**Methods:**

Three polysaccharide fractions (PP I, PP II, PP III) were extracted from *Polygonatum cyrtonema* Hua via ethanol gradient precipitation. The molecular weight distribution and monosaccharide composition were determined using high-performance gel permeation chromatography and pre-column derivatization high-performance liquid chromatography. Hypoglycemic activity was evaluated using insulin-resistant (IR)-HepG2 cells and a db/db mice model. Transcriptomic sequencing and functional enrichment analysis were conducted on PP III-treated db/db mice. Ileocecal short-chain fatty acid contents were quantified by GC-MS. Serum insulin and LEP concentrations were measured via ELISA. Hepatic gluconeogenesis-related gene expression was analyzed using real-time PCR.

**Results:**

PP III (31.0% recovery) exhibited distinct monosaccharide composition dominated by rhamnose and glucose, with molecular weights of 1618.4 Da, 477.4 Da, and 309.5 Da, and demonstrated the most pronounced hypoglycemic activity compared to both other fractions and original PP. In db/db mice, PP III administration significantly reduced hyperglycemia while enhancing insulin sensitivity. Transcriptomic sequencing analysis showed that PP III’s mechanism involves gut microbiota-mediated short-chain fatty acid production (notably acetic acid and propionic acid), subsequently inhibiting hepatic gluconeogenesis. Mechanistic studies identified propionic acid as a key mediator, which may exert its effects by promoting leptin (LEP) secretion and activating the hepatic AMP-activated protein kinase (AMPK) pathway, thus suppressing hepatic gluconeogenesis.

**Conclusion:**

This research identifies PP III as the principal hypoglycemic fraction of PP, providing mechanistic insights and preclinical evidence supporting its potential application as a functional food, health supplement, or pharmaceutical agent for diabetes management.

## 1 Introduction

Diabetes mellitus represents a globally prevalent disease, primarily driven by insulin secretion defects or impaired biological function (1). By 2023, the global diabetic population reached 537 million, with projections indicating a surge to 1.31 billion by 2050; Type 2 diabetes mellitus (T2DM) accounting for 90 to 95% of all clinical cases (2). Most patients necessitate prolonged pharmacotherapy, yet contemporary hypoglycemic agents remain associated with multi-organ toxicity and escalating safety concerns (3).

Traditional Chinese medicine (TCM) contains a rich repository of empirical knowledge in diabetes management, featuring numerous herbs and classical formulations with validated therapeutic function. The identification of safe, high-efficacy, and low-toxicity agents, combined with bioactive components the isolation and mechanistic elucidation, leveraged the unique advantages of TCM in chronic disease intervention. This approach not only fulfills the increasing demand for safer therapeutics but also provides a strategic framework for alleviating diabetes-associated socioeconomic burdens.

*Polygonatum* (Huangjing), first recorded in the *Shennong’s Classic of Materia Medica*, refers to the dried rhizomes of *Polygonatum kingianum* Coll. Et Hemsl., *Polygonatum sibiricum* Red., or *Polygonatum cyrtonema* Hua, which belonging to the *Liliaceae* family. For this study, we specifically used *Polygonatum cyrtonema* Hua, which aligns with its traditional cultivation and use in Zhejiang Province where the plant material was sourced. According to the Chinese Pharmacopoeia (2020 edition), it acts in the spleen, lung, and kidney meridians, with effects of replenishing qi and nourishing yin, invigorating the spleen, moistening the lung, and tonifying the kidney. Traditional applications address spleen and stomach qi deficiency, fatigue, poor appetite due to insufficient stomach yin, dry cough from lung deficiency, chronic hemoptysis, essence-blood depletion, and lumbar-knee soreness. Modern pharmacological investigations confirm its diverse bioactivities, including anti-aging, immunomodulation, glycemic control, and lipid metabolism modulation (4). This multifunctional profile positions *Polygonatum* as a promising candidate for chronic metabolic disorder management.

The phytochemical profile of *Polygonatum* encompasses a variety of bioactive components, primarily polysaccharides, terpenoids, alkaloids, flavonoids, steroids, and volatile oils (5). Particularly noteworthy is *Polygonatum* polysaccharide (PP), which exhibits diverse pharmacological activities encompassing hypoglycemic, antioxidant, and anti-aging activities (6). Our previous study established PP’s capacity to ameliorate multiple diabetic parameters in db/db mice, including significant reductions in fasting blood glucose, urinary glucose, glycated serum protein, and non-esterified fatty acid, as well as improved oral glucose tolerance, enhanced hepatic glycogen synthesis, and increased serum insulin secretion (7). While previous studies have established the hypoglycemic effects of crude *Polygonatum* polysaccharide (PP), the differential bioactivity of specific polysaccharide fractions remains poorly characterized. The present study introduces a systematic ethanol gradient fractionation approach that enables the isolation of distinct polysaccharide fractions with varying bioactivities. While previous studies have established the hypoglycemic effects of crude *Polygonatum* polysaccharide (PP), the differential bioactivity of specific polysaccharide fractions remains poorly characterized. Ethanol gradient precipitation is a classic and gold-standard polysaccharide fractionation method based on the differential solubility of polysaccharides with distinct molecular weights and structural properties in aqueous ethanol systems. Compared with ultrafiltration and column chromatography, this method features mild operating conditions that preserve polysaccharide biological activity, high reproducibility for large-scale fractionation, and low technical barriers (8–10). It has been successfully applied to isolate bioactive polysaccharide fractions from medicinal plants including *Polygonatum sibiricum* (11) and Schisandra chinensis (12). The present study introduces a systematic ethanol gradient fractionation approach optimized for *Polygonatum cyrtonema* Hua that enables the isolation of distinct polysaccharide fractions with varying bioactivities. This strategy provides new insights into structure-activity relationships that cannot be obtained from crude polysaccharide extracts alone.

To further optimize therapeutic efficacy, we implemented a systematic ethanol gradient fractionation of *Polygonatum cyrtonema* Hua aqueous extract, yielding three distinct components: PP I, PP II and PP III. This study investigates the hypoglycemic effects and underlying mechanisms of these fractions through both *in vitro* and *in vivo* experiments.

## 2 Materials and methods

### 2.1 Fractionated extraction and characterization

#### 2.1.1 Ethanol gradient extraction

Fresh rhizomes from 3-year-old *Polygonatum cyrtonema* Hua were obtained from Zhanfei Family Farm (Jiangshan City, Zhejiang Province, China). The plant material was authenticated by Professor Wangping (College of Pharmacy, Zhejiang University of Technology), and voucher specimens (Lot No. 20231106) were deposited in our laboratory. Following previously established protocols for *Polygonatum* polysaccharide extraction (7), exactly 1.0 kg of *Polygonati* Rhizoma (dried rhizome of *Polygonatum cyrtonema* Hua) was selected, thoroughly washed, oven-dried at 45 °C to constant weight, and pulverized to pass through a 40-mesh sieve, then defatted by soaking in 80% ethanol. The residue then underwent sequential hot water extraction. The combined aqueous extract was clarified by filtration. Polysaccharides were fractionated by stepwise ethanol precipitation: the extract was brought to 40%, 60%, and finally 80% ethanol concentration. Each step involved overnight precipitation at 4 °C, followed by centrifugation and vacuum drying, yielding the precipitates designated as PP I, PP II, and PP III, respectively. All fractions were stored in a desiccator at 4 °C until use.

#### 2.1.2 Structural characterization

The molecular weight distribution was determined using high-performance gel permeation chromatography (HPGPC) with a RID-20 differential detector and a gel column system comprising TSK gel G3000PWXL, G4000PWXL, and G5000PWXL (7.8 mm × 300 mm). Monosaccharide composition was analyzed by pre-column derivatization high-performance liquid chromatography (HPLC). PP samples were hydrolyzed with trifluoroacetic acid, and the hydrolysate was concentrated under reduced pressure with methanol to remove trifluoroacetic acid. Derivatization was performed using NaOH and 1-phenyl-3-methyl-5-pyrazolone (PMP) in methanol, followed by HPLC analysis.

### 2.2 Glucose consumption assay in HepG2 cells

#### 2.2.1 Insulin resistance model establishment

HepG2 cells in the logarithmic growth phase were prepared in DMEM high-glucose culture medium (5 × 10^5^ cells/mL) and seeded into 96 well plates (100 μL/well). After 24 h of incubation, cells were treated with insulin gradients (0–1600 nmol/L) in DMEM for 24 h, followed by additional incubation with 100 nmol/L insulin for another 24 h. Glucose content in the supernatant was then measured using glucose determination kit (Shanghai Rongsheng Biotech Co. Ltd, Shanghai, China), with parallel cell viability assessment via CCK8 assay.

#### 2.2.2 Effects on IR-HepG2 glucose consumption

Insulin-resistant HepG2 cells (5 × 10^5^ cells/well in 12-well plates) were treated with PP fractions (0–500 mg/L) and with or without 100 nmol/L insulin (24 h). Glucose consumption was measured using the glucose oxidase method, and glucose consumption was calculated by subtracting the glucose content of blank wells.

### 2.3 Mice experiments

Male db/db mice at eight weeks of age (35–40 g) and m/m littermate controls (Jiangsu Changzhou cavens laboratory animal Co., Ltd) were housed under SPF conditions at 25 ± 2 °C at Zhejiang Academy of Traditional Chinese medicine. Following a 7-day adaptive feeding, db/db mice were randomly assigned to model (DM), PP I (1.0 g/kg), PP II (1.0 g/kg), PP III (1.0 g/kg), and metformin positive control groups (n=8) for 8 weeks. Mice in normal controls (NC) and DM groups received water by gavage. Body weight was recorded biweekly, and fasting blood glucose (tail vein sampling) was measured weekly. At the end of the experiment, blood was collected from the femoral artery under anesthesia, and serum insulin and LEP levels were measured by ELISA.

### 2.4 Transcriptome sequencing and data processing

#### 2.4.1 Total RNA extraction

Total RNA was extracted from five liver tissue samples each of the groups, using TRIzol^®^ reagent. The lysates were mixed with 300 μL of chloroform/isoamyl alcohol (24:1), vortexed vigorously, and centrifuged at 12,000×g for 8 minutes at 4 °C. The upper aqueous phase was transferred to a new tube, mixed with 2/3 volume of isopropanol, gently inverted, and centrifuged at 17,500×g for 25 minutes at 4 °C. The supernatant was discarded, and the RNA pellet was washed with 0.9 mL of 75% ethanol at 4 °C for 3 minutes at 17,500×g. After air-drying for 3–5 minutes, the RNA was dissolved in 50 μL of DEPC-treated water.

#### 2.4.2 Library construction

The BGI Optimal Series mRNA Library Construction Kit (BGI-Shenzhen, China) was used. RNA samples were denatured to disrupt secondary structures, and mRNA was enriched using oligo(dT) magnetic beads. First-strand cDNA was synthesized using random hexamer primers and reverse transcriptase. Second-strand synthesis was performed in a reaction mixture containing dUTP. The double-stranded cDNA was heat-denatured, circularized using a ligation system, and digested with exonuclease to remove linear DNA fragments. The resulting single-stranded circular DNA (DNA nanoballs, DNBs) was amplified via rolling circle replication and loaded onto a high-density sequencing chip. Paired-end 150-bp sequencing was conducted on a T7 sequencer (BGI-Shenzhen, China) using combinatorial probe-anchor synthesis (cPAS) technology.

#### 2.4.3 Bioinformatics

Raw sequencing data were filtered using SOAPnuke v2.2.1. to remove adapter-contaminated reads, Reads containing more than 5% unknown bases (N), and Low-quality reads (≥20% of bases with quality scores <15). Clean data were analyzed using Dr.Tom multi-omics platform (https://biosys.bgi.com) for downstream processing, visualization, and mining. Differential gene expression was analyzed using Phyper, followed by Gene Ontology (GO; http://www.geneontology.org/) and Kyoto Encyclopedia of Genes and Genomes (KEGG; https://www.kegg.jp/) enrichment analysis with a significance threshold of Q-value ≤0.05. Raw sequencing data were filtered using SOAPnuke v2.2.1 to remove adapter-contaminated reads, reads containing more than 5% unknown bases (N), and low-quality reads (≥20% of bases with quality scores < 15).

### 2.5 SCFA targeted metabolomic assays

SCFA contents (acetic acid, propionic acid, isobutyric acid, butyric acid, isovaleric acid, valeric acid, isohexanoic acid and hexanoic acid) in ileocecal contents were quantified by GC-MS after n-butanol extraction.

### 2.6 Real-time PCR analysis

Hepatic RNA were measured using a Nano Drop spectrophotometer (ND-2000, NanoDrop Technologies, USA). Synthesis of cDNA (PrimeScript RT Master Mix kit) and real-time PCR (SYBR Premix Ex Taq kit) was performed as described. The relative expression (2^-ΔΔCt^ method) was normalized to *Ef-1a*. The primers listed in **Table 1**.

**Table 1:** Primer sequences for real-time PCR analysis.

Gene	Primer direction	Sequences
*Adra1a*	Forward	CGGAGGATGAGACCATCTGC
	Reverse	TGACTTGTCGGTCTTGAGGC
*Lkb1*	Forward	GCCCACCACTCTCTGACCTACTC
	Reverse	CTGTGCTGCCTAATCTGTCGGATG
*Ampk*	Forward	CGAGTGTTCGGAGGAGGAGGTC
	Reverse	GTGGGCTGGTTGCTAGGTAGAAATC
*Torc2*	Forward	CCCTACCTGACCTCACCAACCTAC
	Reverse	CAGACCTCCACTGATGCCCAAATG
*Creb*	Forward	ACTCAGCCGGGTACTACCAT
	Reverse	ACGCCATAACAACTCCAGGG
*Pepck*	Forward	ATGGGGTGTTTGTAGGAGCA
	Reverse	CCGAAGTTGTAGCCGAAGAA
*Ef-1a*	Forward	CGAGCCACCATACAGTCAGA
	Reverse	CCATTCCAACCAGAAATTGG

### 2.7 Ethics approval

All animal experimental procedures were approved by the Laboratory Animal Welfare Ethics Committee of the Zhejiang Academy of Traditional Chinese Medicine (Approval No [2024]:024). All methods were performed in accordance with relevant guidelines and regulations.

### 2.8 Statistical analysis

Data are presented as mean ± standard deviation (SD). Prior to statistical analysis, the normality of data distribution was confirmed using the Shapiro-Wilk test, and homogeneity of variance was verified using Levene’s test. One-way analysis of variance (ANOVA) followed by LSD and SNK *post-hoc* tests was performed using SPSS Statistics 21.0. Differences were considered statistically significant at *p* < 0.05.

## 3 Results and discussion

### 3.1 Characterization of ethanol-fractionated PP extracts

**Figure 1:**
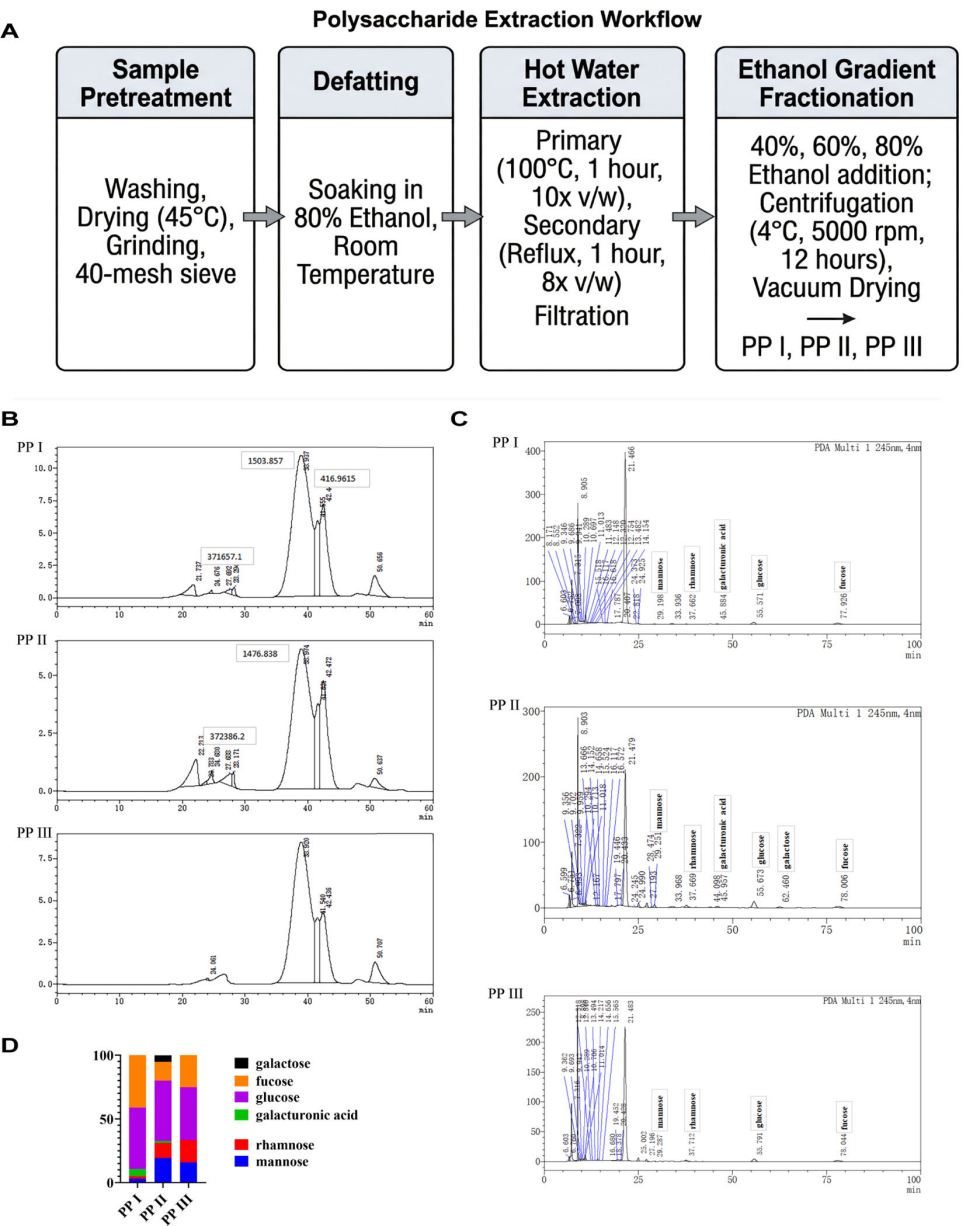
Extraction and detection of PP from different parts. **(A)** Linear stepwise schematic of ethanol gradient extraction and fractionation of PP; **(B)** The molecular weight of PP was determined using HPGPC; **(C)** Monosaccharide composition was analyzed by pre-column derivatization high-performance liquid chromatography; **(D)** Column chart of monosaccharide composition.

Three PP fractions were successfully isolated *via* ethanol precipitation, with recovery rates of 3.7% (PP I), 2.0% (PP II), and 31.0% (PP III) (**Figure 1A**). The molecular weights analysis revealed similar patterns across fractions: PP I (1610.1 Da, 470.7 Da, 309.3 Da), PP II (1582.4 Da, 456.4 Da, 306.0 Da), and PP III (1618.4 Da, 477.4 Da, 309.5 Da) (**Figure 1B**). Monosaccharide composition analysis identified mannose, rhamnose, glucose and fucose as primary constituents (**Figure 1C**), with glucose, fucose and rhamnose predominating. Notably, PP III lacked galacturonic acid and galactose, and as the ethanol concentration increased, fucose content decreased while rhamnose content increased (**Figure 1D**).

### 3.2 *In vitro* hypoglycemic activity

**Figure 2:**
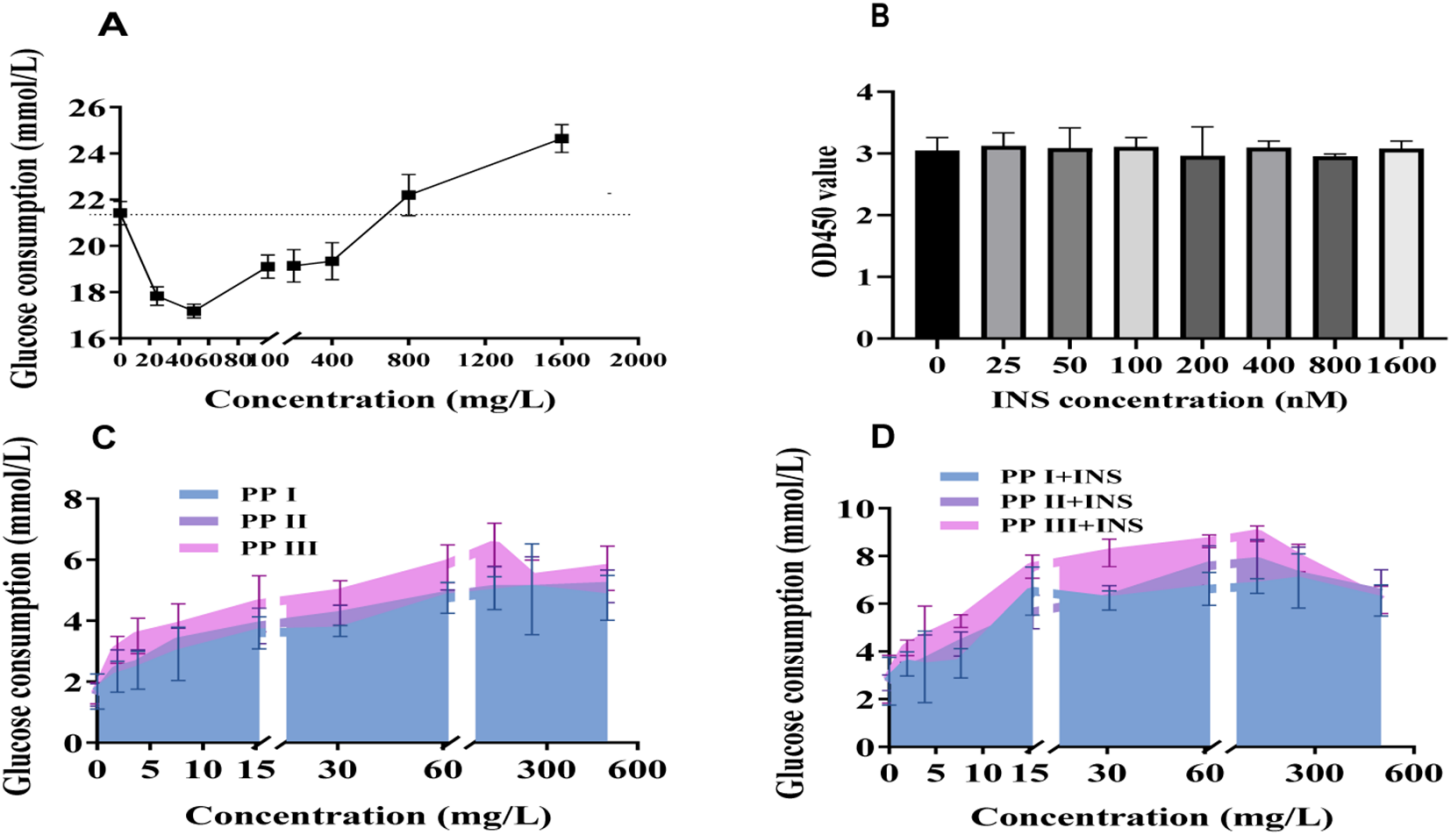
*In vitro* hypoglycemic effects of different extracts of PP. **(A)** Glucose consumption in HepG2 cells treated with different PP fractions; **(B)** Viability of HepG2 cells treated with insulin gradients determined by CCK-8 assay; **(C)** Glucose consumption in insulin-resistant (IR)-HepG2 cells treated with PP fractions; **(D)** Effects of PP Extract on glucose consumption in IR-HepG2 cells with insulin.

Insulin resistance modeling in HepG2 cells identified 800 nmol/L and 1600 nmol/l insulin as optimal concentrations for inducing resistance without cytotoxicity (**Figures 2A, B**). Dose-response assays (0–600 mg/L) demonstrated concentration-dependent glucose consumption enhancement across all PP fractions, with PP III exhibiting the most pronounced efficacy (**Figure 2C**). Insulin sensitivity assays confirmed PP III’s superiority in restoring insulin responsiveness (**Figure 2D**), suggesting its potential as the most potent hypoglycemic candidate.

### 3.3 *In vivo* hypoglycemic efficacy

**Figure 3:**
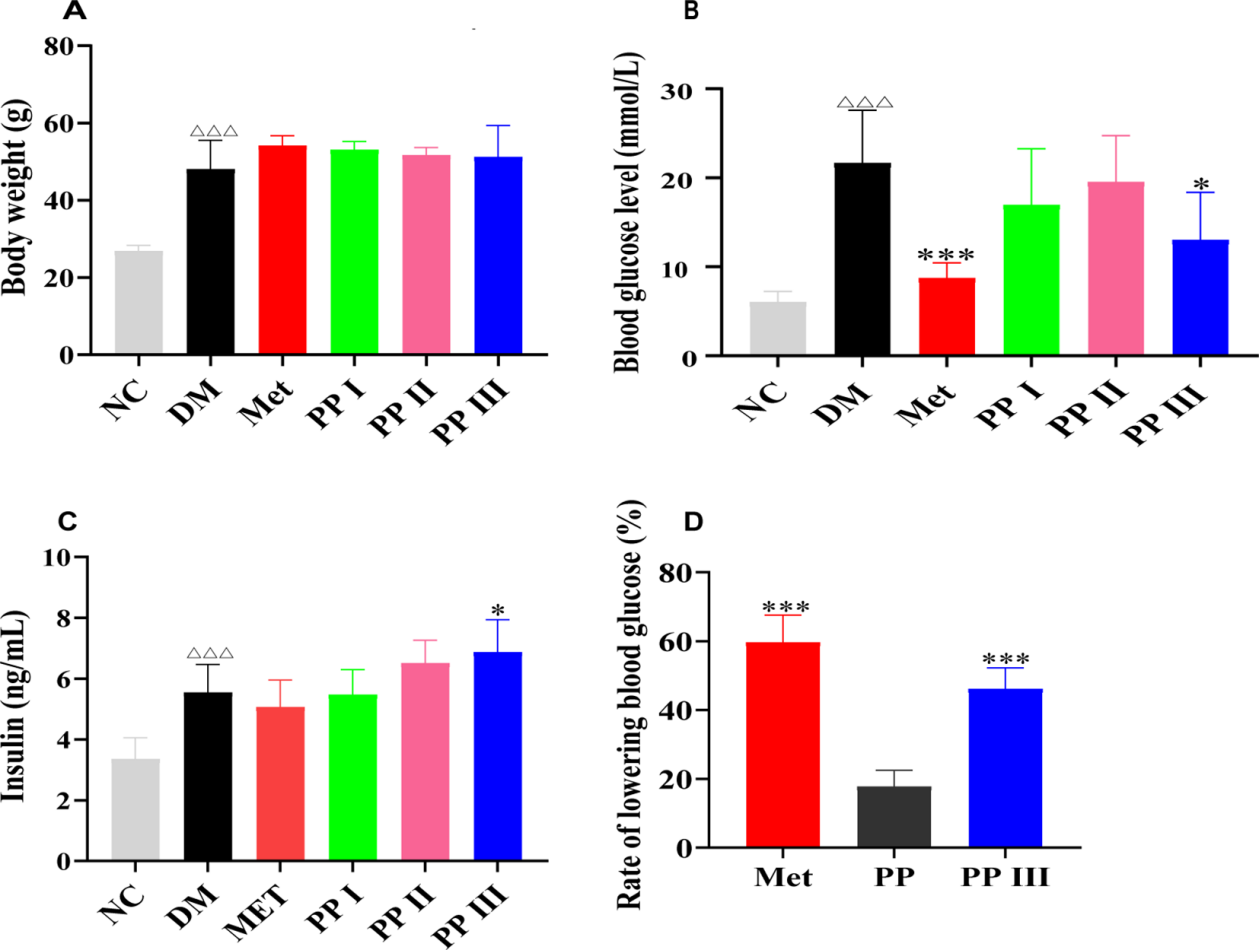
Hypoglycemic effect of PP Extracts *in vivo*. **(A)** Body weight; **(B)** Blood glucose level; **(C)** Insulin level in serum; **(D)** Rate of lowering blood glucose. Data=Mean ± SEM. ^△△△^P < 0.001 versus NC; *P < 0.05, **P < 0.01, ***P < 0.001 versus DM.

In db/db mice, PP III administration induced the most significant glycemic reduction among all treatment groups, despite comparable body weight across groups (**Figures 3A, B**). Furthermore, serum insulin analysis showed that PP III had a unique ability to significantly increase serum insulin levels (**Figure 3C**), which was superior to the effects of pre-fractionated PP (7) and the other two fractions (**Figure 3D**). The metformin positive control group (150 mg/kg/day) demonstrated expected antihyperglycemic effects, reducing fasting blood glucose by approximately 35% compared to the diabetic model group. Notably, PP III treatment (1.0 g/kg/day) achieved comparable glycemic control to metformin, with no statistically significant difference between these two groups (*p* > 0.05), while both treatments showed significant improvement over the model group (*p* < 0.001).

### 3.4 PP III modulates SCFA and LEP in db/db mice

**Figure 4:**
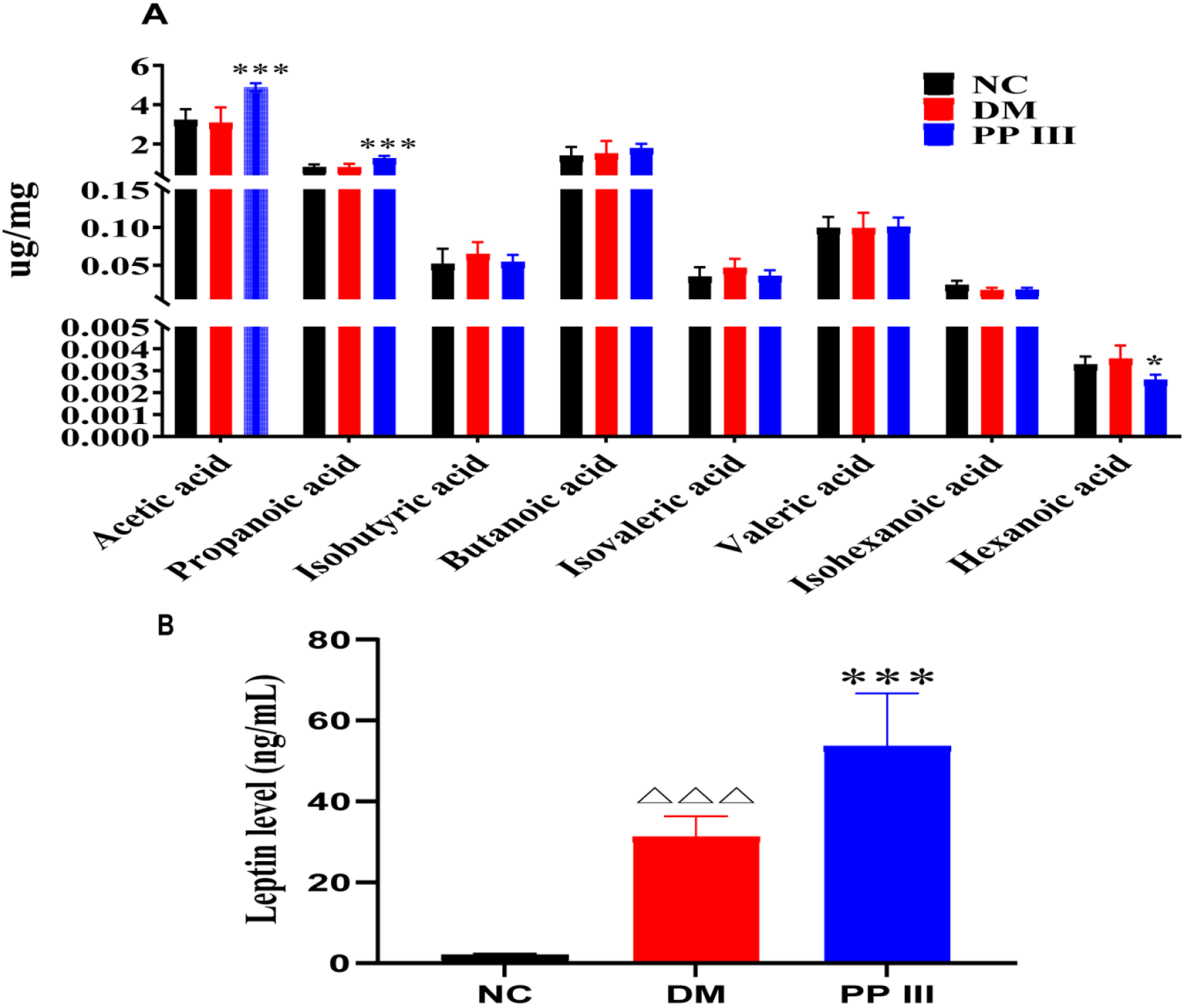
Effects of PP III on SCFA in intestinal contents of db/db mice. **(A)** LEP level in serum; **(B)** SCFA level in intestinal contents. Data=Mean ± SEM. ^△△△^P < 0.001 versus NC; *P < 0.05, ***P < 0.001 versus DM.

Polysaccharides modulate host metabolome by influencing the composition and function of the gut microbiota, thereby exerting various biological effects (12). In this study, we investigated the SCFA metabolome in the ileocecal contents of db/db mice. Targeted metabolomic analysis showed that PP III exerted a dual regulatory effect on SCFAs: it significantly increased acetic acid (AA) and propionic acid (PA) levels, while markedly decreasing hexanoic acid (HA) content (**Figure 4A**). Previous studies have shown that decreased abundance of AA and PA is associated with the progression of T2DM (13). Additionally, clinical research has indicated that improved glycemic control in T2DM patients correlates with elevated AA levels (14). Exogenously administered AA has shown potential in mitigating obesity-associated T2DM in both rats (15) and humans (16).

While colonic SCFA enhancement has demonstrated metabolic improvement in T2DM rats (17), our findings uniquely identify ileocecal SCFA enrichment as PP III’s distinctive mechanism. These observations suggest that PP III may regulate glucose metabolism and insulin sensitivity through modulating SCFA production, which in turn influence the gut microbiota and host metabolic pathways. This mechanism further supports the potential of PP III as a dietary intervention for managing diabetes and related metabolic disorders.

These results position SCFAs as critical targets of PP III’s antidiabetic activity. Though their precise mechanism of action in hepatic gluconeogenesis remains to be fully elucidated, PA has been shown to stimulate LEP secretion from adipocytes, thereby activating liver AMPK. Our results showed that serum leptin (LEP) expression was significantly upregulated in the PP III group (p < 0.001) (**Figure 4B**), which was likely driven by SCFAs such as PA. The data presented reinforce the significance of SCFA as key mediators in gut-liver metabolic communication, providing a promising avenue for future research into polysaccharide-based therapeutic strategies targeting gut health and metabolic regulation in diabetes management.

### 3.5 Transcriptomic and functional enrichment analysis of PP III in db/db mice liver

**Figure 5:**
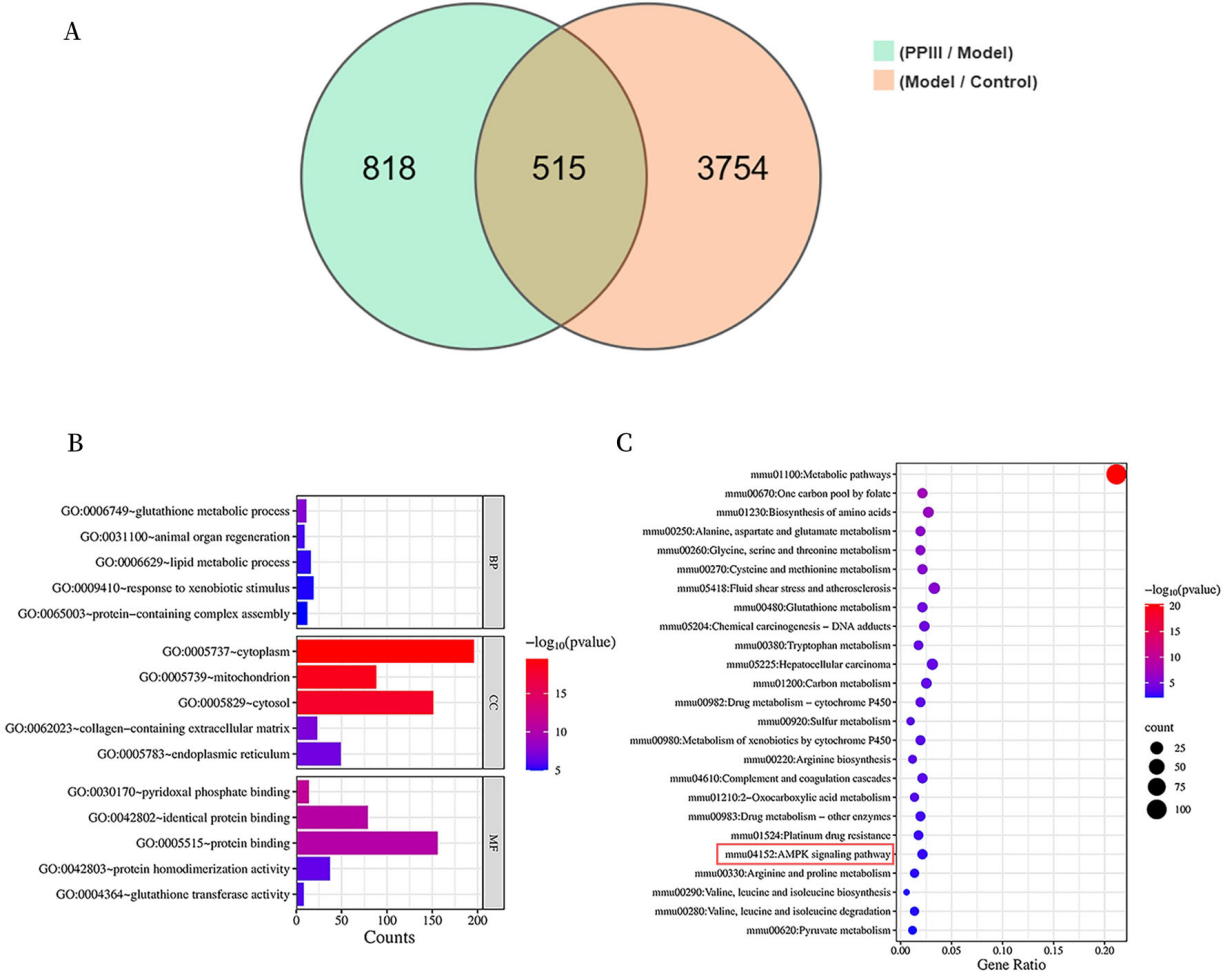
Transcriptomic and Functional Enrichment Analysis of PP III in db/db mice Liver. **(A)** Venn diagram of differentially expressed genes among three groups; **(B)** Enrichment analysis of GO function; **(C)** Enrichment analysis of KEGG pathway.

Compared with the NC group, the DM group exhibited 2,116 upregulated and 2,153 downregulated genes. In the PP III group versus the DM group, Transcriptomic analysis identified 579 upregulated and 754 downregulated genes. Venn diagram analysis (**Figure 5A**) identified shared and unique differentially expressed genes across the three groups. Enrichment analysis of 515 differentially expressed genes revealed significant GO terms in biological processes (e.g., glutathione metabolism, lipid metabolism), cellular components (e.g., cytoplasm, mitochondria), and molecular functions (e.g., glutathione transferase activity) (**Figure 5B**). KEGG pathway analysis identified 55 enriched pathways, including amino acid metabolism, glutathione metabolism, and AMPK signaling pathways (**Figure 5C**). Top 25 most significantly enriched pathways are visualized in the bubble plot (**Figure 5C**).

### 3.6 Effects of PP III on the mRNA of gluconeogenesis-related pathways in db/db mice

As depicted in **Figure 5**, PP III increased the expression of genes related to gluconeogenesis, including ADRA1A, LKB1, and AMPK in db/db mice, while reducing the expression of genes such as TORC2, CREB, and PEPCK. Among them, significant differences were observed in the expression levels of ADRA1A, AMPK, CREB, and PEPCK compared with the DM group (P<0.05). These results indicate that PP III may inhibit gluconeogenesis through regulating the genes associated with the ADRA1A-AMPK-TORC2 pathway, which in turn contributing to the reduction of blood glucose levels.

**Figure 6:**
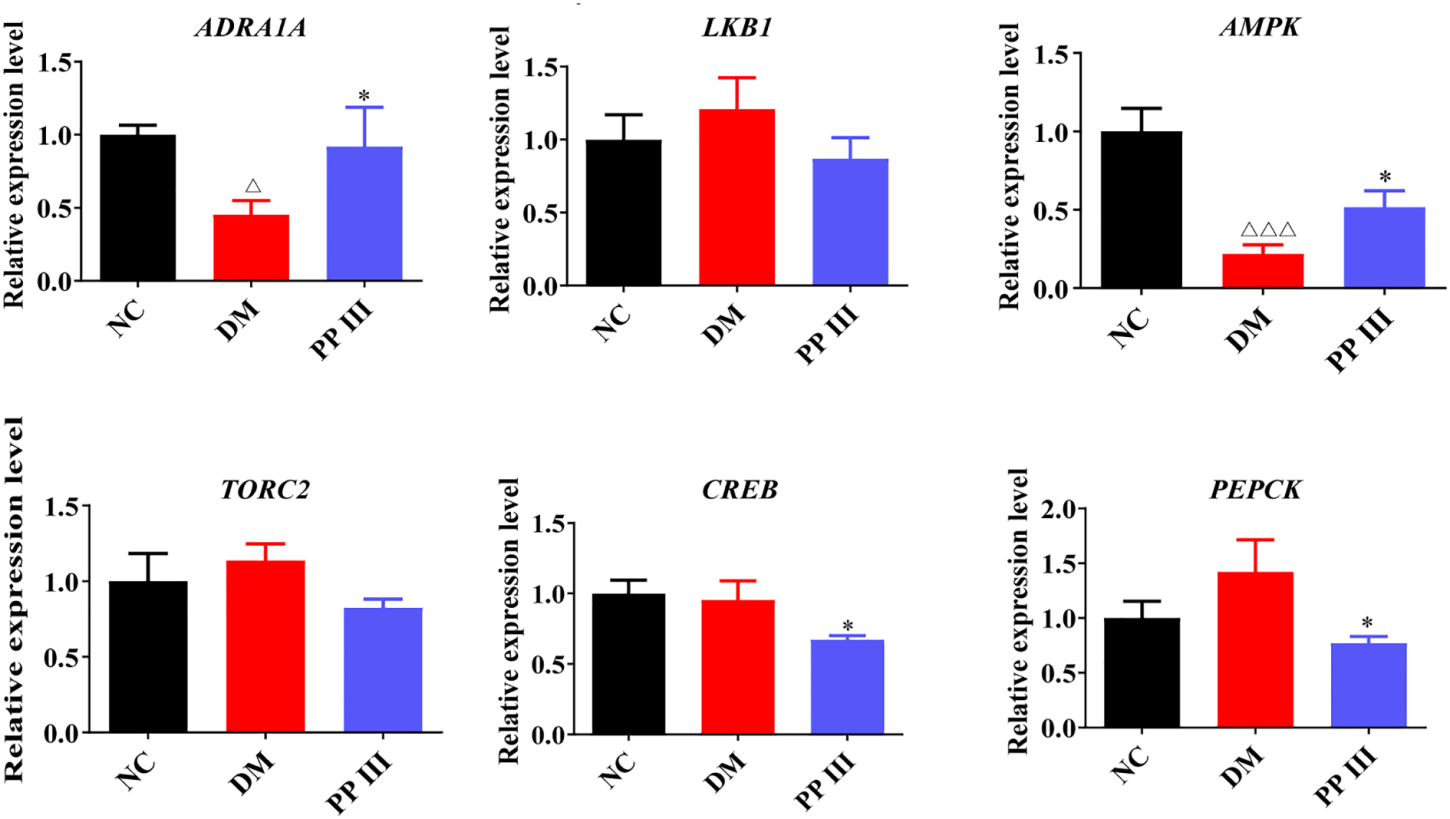
Effects of PP III on the mRNA of Gluconeogenesis-related Pathways in db/db mice. Data=Mean ± SEM. ^△^P < 0.05, ^△△△^P < 0.001 versus NC; *P < 0.05, versus DM.

Abnormally elevated hepatic gluconeogenesis plays a critical role in the pathogenesis of diabetes. The liver is central to glucose metabolism (18), predominantly regulated by insulin and glucagon. In the context of hepatic insulin resistance, gluconeogenesis is upregulated while glycogen synthesis is reduced and glycogenolysis enhanced, collectively contributing to hyperglycemia. In T2DM, excessive gluconeogenesis leads to increased endogenous glucose production, a hallmark of its pathophysiology (19). This process is primarily driven by key enzymes, especially phosphoenolpyruvate carboxykinase (PEPCK) and glucose-6-phosphatase (G6Pase) (20). Our study evaluated the regulatory effects of PP III on these enzymes. In db/db mice, the expression of PEPCK was significantly upregulated compared to normal mice. Treatment with PP III markedly downregulated the expression of PEPCK (p<0.05) (**Figure 6**), demonstrating that PP III effectively suppresses key gluconeogenic enzymes to reduce blood glucose levels.

The expression of enzymes such as PEPCK, which are crucial for gluconeogenesis, is finely regulated by the AMPK-TORC2 pathway (21). AMPK can phosphorylate the Ser171 site of TORC2, causing it to stay in the cytoplasm and reducing its binding with CREB in the nucleus, thus inhibiting the transcription of PEPCK (22, 23). Consequently, TORC2 is regarded as the “molecular switch” for regulating gluconeogenesis (24). However, in the state of insulin resistance, the elevated intracellular cAMP level leads to the dephosphorylation of TORC2, followed by its translocation to the nucleus, where it binds to CREB, promotes the expression of key enzymes of gluconeogenesis, and ultimately exacerbates the gluconeogenesis process (25). This study examined the regulatory effect of PP III on the ADRA1A-AMPK-TORC2 pathway in the liver of db/db mice. Nevertheless, it had a relatively minor impact on LKB1. These findings suggest that PP III may inhibit the regulation of the expression of key downstream gluconeogenic enzymes and block the abnormally active state of gluconeogenesis by increasing the levels of ADRA1A and AMPK. This mechanism further supports the application value of PP III as a potential intervention drug, which can be used to improve metabolic abnormalities in T2DM. Previous studies have confirmed that a variety of traditional Chinese medicines and their active ingredients can exert hypoglycemic effects through this pathway (18).

It should be noted that this study focused on polysaccharide components, while *Polygonatum cyrtonema* Hua contains a variety of natural compounds in glycosylated forms (e.g., flavonoid glycosides, steroidal saponins, phenolic glycosides) (5), and their aglycone moieties are the core pharmacophores responsible for the hypoglycemic activity of these glycosylated compounds (26, 27). The Sugar moieties in glycosylated compounds primarily enhance water solubility, facilitating intestinal absorption, and aid targeted delivery, whereas the aglycones directly bind to key molecular targets of glucose metabolism (e.g., AMPK, PPARγ, SGLT2, PEPCK) to regulate glycemic homeostasis (28). For example, flavonoid aglycones (quercetin, kaempferol) from *Polygonatum* can activate the hepatic AMPK pathway to inhibit gluconeogenesis, and steroidal saponin aglycones (diosgenin) can regulate gut microbiota to increase SCFA production and improve insulin resistance (29). Although ethanol gradient precipitation effectively enriched the polysaccharide fraction PP III, trace amounts of glycosylated non-polysaccharide components may coexist in PP III, and their aglycone moieties can be released by gut microbiota enzymatic hydrolysis in the ileocecum (12, 26). Thus, the pronounced hypoglycemic activity of PP III observed in this study may be a synergistic effect of the polysaccharide itself and trace aglycone moieties, rather than a single-component effect, a phenomenon that has also been reported in other medicinal plant polysaccharide studies (30), where polysaccharides and aglycones act synergistically to enhance antidiabetic activity. This perspective does not negate the core finding that PP III is the principal hypoglycemic fraction of PP, but provides a more comprehensive interpretation of the experimental results: the antidiabetic effect of *Polygonatum cyrtonema* Hua is a result of the synergistic action of multiple bioactive components. Future studies will use UHPLC-MS/MS to isolate and identify aglycone moieties in PP III, verify their individual hypoglycemic activity via insulin-resistant HepG2 cells and db/db mice models, and quantify their synergistic effect with PP III using the Chou-Talalay combination index (CI) method. In addition, the gut microbiota-mediated metabolic transformation of aglycone moieties will be further investigated to clarify their precise contribution to the hypoglycemic activity of PP III.

## 4 Conclusions

In conclusion, PP III exhibited the most pronounced hypoglycemic effects among the tested fractions and showed superior efficacy to the original PP. *In vitro* studies revealed that PP III significantly enhanced glucose uptake and improved insulin sensitivity in insulin-resistant HepG2 cells. *In vivo* experiments using db/db mice further confirmed these findings, with PP III administration effectively reducing blood glucose levels while increasing serum insulin concentrations. Notably, PP III treatment increased short-chain fatty acid (SCFA) levels, particularly acetic acid and propionic acid, in the ileocecal contents of db/db mice. These metabolic changes were associated with increased leptin (LEP) expression and suppressed hepatic gluconeogenic pathways. Collectively, our results demonstrate that PP III exerts its antidiabetic effects through multiple mechanisms, highlighting its potential as a novel therapeutic candidate for diabetes management.

## Data Availability

The datasets presented in this study can be found in online repositories. The names of the repository/repositories and accession number(s) can be found in the article/supplementary material.
